# Wuhan College Students’ Self-Directed Learning and Academic Performance: Chain-Mediating Roles of Optimism and Mental Health

**DOI:** 10.3389/fpsyg.2021.757496

**Published:** 2022-01-24

**Authors:** Jun Li, Dong Yang, Ziao Hu

**Affiliations:** ^1^Department of Education Management, China-Asean International College, Dhurakij Pundit University, Bangkok, Thailand; ^2^Suryadhep Teachers College, Rangsit University, Pathumthani, Thailand

**Keywords:** self-directed learning, academic performance, optimism, mental health, Wuhan

## Abstract

This study explored the chain-mediating roles of optimism and mental health in the relation of self-directed learning with academic performance among college students in Wuhan during long-term online teaching. In total, 473 valid responses were obtained from students at three Wuhan universities. Self-directed learning, optimism, mental health, and academic performance scales were used as measurement instruments; a 5-point Likert scale was employed for all items. To examine the instruments’ reliability and validity, a measurement model was constructed; moreover, structural models were employed for assessing the chain mediation model. This study confirmed that self-directed learning was a positive predictor of academic performance in Wuhan college students. Optimism and mental health were two mediators and partially jointly mediated the relation of self-directed learning with academic performance. The results revealed that self-directed learning only partially positively predicted academic performance. The aforementioned relationship was partially mediated by optimism and mental health, highlighting the essential roles of optimism and mental health in the learning and learning outcomes of Wuhan college students.

## Introduction

As a global public health emergency, the COVID-19 pandemic has resulted in an increase in university Students’ perception of stress (Husky et al., 2020). Facing the double pressure of the sudden pandemic and online learning, college students may have become more susceptible to anxiety and pressure (Goldmann and Galea, 2013; Byrnes et al., 2020; Safa et al., 2021). In February 2020, at the beginning of the epidemic that originated in Wuhan, China’s Ministry of Education issued “Instructions for Adequate Implementation of Online Education Organization and Management in Ordinary Colleges and Universities during Pandemic Prevention and Control,” requesting joint implementation and protection of online teaching to achieve class suspension without interrupting learning (Ministry of Education of the People’s Republic of China, 2020). Wuhan in 2020 was one of the regions hardest hit by the pandemic. Thus, Wuhan college students have experience in online education for a relatively extended period, and their self-directed learning skills may have been trained and improved through the online teaching model since the 2020 year (Li, 2020; Zhang W. et al., 2020; Zhang X. et al., 2020; Zhang Z. et al., 2020). Therefore, Wuhan college students were selected as the research targets.

Given the impact of COVID-19, online teaching is likely to become a more prevalent teaching mode (Jaberzadeh and Mansouri, 2021; Yang et al., 2021). Studies have revealed that, in the context of long-term online teaching and the school closure policy, college Students’ self-directed learning ability is instrumental to the smooth development of online teaching (Cai et al., 2020; Xiang et al., 2021). Self-directed learning refers to learning with spontaneity and purposefulness that allows for self-management and self-evaluation (Holec, 1979). The level of college Students’ self-directed learning also influences the future development and spread of online education (Xie et al., 2020). In the constructivist view, the importance of student-centered autonomous exploration and knowledge construction is emphasized (Piaget, 1980); self-determination theory suggests that targeted and active learning behaviors can generate more favorable learning outcomes (Deci and Ryan, 1985). According to survey-based research, self-directed learning has a strong positive effect on learners’ academic performance (Zhou et al., 2010; Kim, 2017; Oducado, 2021). Therefore, this research posited that self-directed learning is a significant positive predictor of the academic performance of Wuhan college students.

Pandemic-induced changes in learning style can cause stress and negative emotions. Studies have revealed that optimism help individuals adapt to stressful life events (Williams, 2003; Yu and Zheng, 2011). According to self-determination theory and psychological capital theory, self-determined behaviors can produce more positive and optimistic emotions. In psychological capital theory, optimism—a type of psychological capital—is described as a quality that drives individuals to achieve better outcomes and performance (Deci and Ryan, 1985; Luthans et al., 2015). Studies on self-directed learning, optimism, and academic achievement have indicated that individuals who are adept at self-directed learning are more likely to experience the positive emotions of satisfaction and optimism (Elliot and Dweck, 1989) and that optimists are more likely to adopt effective coping strategies when facing stressful academic scenarios (Piko et al., 2013; Bai and Xie, 2018), thereby maintaining individual academic outcomes (El-Anzi and Owayed, 2005; Wang et al., 2011). Therefore, this study posited that self-directed learning generates optimistic emotions and affects the academic achievement of college students in Wuhan.

The experience of being in an environment in which many negative life events are occurring during the pandemic can jeopardize an individual’s mental health and further trigger a psychological crisis (Zhang W. et al., 2020; Zhang X. et al., 2020; Zhang Z. et al., 2020). The pandemic-induced shifts in teaching and learning modes may have resulted in considerable challenges and psychological pressure on Wuhan college students without online learning experience (Clabaugh et al., 2021; Jin et al., 2021). According to ecological systems theory and self-determination theory, individuals who employ initiative in learning are more likely to meet their own expectations as well as those of their parents and society, thereby obtaining positive emotional feedback and support; such positive support can improve the learner’s mental health, which in turn provides support for academic achievement through a healthy psychological state (Maslow, 1954; Bronfenbrenner, 1992; Ryan and Deci, 2000; Luthans and Youssef, 2007). Studies on self-directed learning, mental health, and academic achievement have reported that, relative to those of individuals with lower learning initiative, the mental health levels of individuals with higher learning initiative were generally more favorable (Tuffin et al., 2001; Chen, 2015), and those with more favorable mental health are typically more effective at adjusting to academic pressure to maintain the positive development of their academic performance (Bostani et al., 2014; Zhao, 2020). Thus, this study held that self-directed learning affects Wuhan college Students’ mental health and subsequently their academic performance.

Studies assessing the relation between optimism and mental health have highlighted optimism as a major predictor of an individual’s mental health (Hanssen et al., 2014), and it is generally regarded as having a psychologically protective role in mental health (Chang, 2002). Optimism can help people withstand the impact of academic pressure on mental health (Zhang et al., 2011). Therefore, both optimism and mental health may mediate the connection of self-directed learning with academic achievement; moreover, optimism and mental health may have a chain-mediating relationship. When viewed collectively, self-determination theory and ecological systems theory suggest that (1) autonomous and spontaneous learning can generate positive emotions and expectations toward learning, (2) positive emotions can help maintain an individual’s mental health, and (3) a favorable psychological state is instrumental in maintaining the highly efficient learning outcomes of an individual (Maslow, 1954; Bronfenbrenner, 1992; Ryan and Deci, 2000; Luthans and Youssef, 2007). Empirical research has similarly revealed that autonomous and spontaneous learning behaviors may induce an optimistic emotional experience in learners (Alkhedr, 1999; El-Anzi and Owayed, 2005), and optimistic individuals are particularly adept at maintaining a relaxed state that allows them to resist academic stress (Zhang et al., 2011; Hanssen et al., 2014), thereby ensuring highly efficient learning outcomes (Bostani et al., 2014; Agnafors et al., 2020). On the basis of the preceding discussion, Wuhan college students were selected as the research targets for exploring the relation of self-directed learning with college performance; the chain-mediating roles of optimism and mental health between self-directed learning and performance were also examined. In this manner, the effect that self-directed learning has on the internal relationship mechanism of Wuhan college Students’ academic performance was investigated.

### Self-Directed Learning and Academic Performance

A central tenet of humanistic learning theory, with psychologist Carl Rogers as the representative, is the importance of people, self-concept, and emotional factors in the learning process; thus, helping students acquire the ability to learn is more central than teachers’ knowledge delivery (Rogers, 1983). Constructivist theory holds that learning is student-centered, and it should emphasize learners’ active exploration of knowledge, their spontaneous discovery, and their active construction of the meaning of the knowledge they have acquired (Piaget, 1980). In the triarchic theory of intelligence, the crucial role of individual initiative in the construction of cognitive structure is similarly emphasized (Surhone et al., 2010). Therefore, student-centered as well as autonomous and spontaneous learning behavior is what education strives toward. In the education field, spontaneous learning is also called self-directed learning (Knowles, 1975; Lemmetty and Collin, 2020). Holec (1979) defined self-directed learning as an individual’s ability to be responsible for their own learning and specifically explained it as the ability of a learner to define their particular learning objectives, content, and progress as well as to select learning methods and techniques, track the learning process, and assess the learning results. Self-directed learning involves a learner’s desire and ability to independently make choices regarding their own learning (Littlewood, 1999). Self-determination theory suggests that individuals have a natural inclination to act on their own willpower. Therefore, people are more motivated by what they wish to do than by what they are required to do (Deci and Ryan, 1985). According to this theory, after learners have independently established their learning goals, the adoption of self-monitoring-based active learning behaviors can lead to greater learning results. Autonomous and independent learning are critical methods for college students given their adult status (Tough, 1989; Loeng, 2020). Khiat (2015) revealed that adult Students’ level of perceptive skills regarding indices of self-directed learning directly or indirectly affects their academic performance. A model constructed by Kim (2017) similarly indicated that, in path analysis, self-directed learning can directly affect academic achievement; thus, self-directed learning plays a positive role in college Students’ academic achievement (Cazan and Schiopca, 2014; Oducado, 2021). Therefore, on the basis of the theoretical perspectives and literature support provided in the preceding section, Hypothesis 1 was formulated as follows:

H1: Self-directed learning positively predicts academic performance.

### Self-Directed Learning, Optimism, and Academic Performance

Scheier defined optimism as a positive expectation of future results (Scheier and Carver, 1985). When viewed collectively, self-determination theory and psychological capital theory suggest that self-determination behavior can generate positive and optimistic emotions; as a type of psychological capital, optimism is described as a trait that motivates an individual to achieve more favorable outcomes and performance (Deci and Ryan, 1985; Luthans et al., 2015). During self-directed learning, learners generate positive emotions and expectations of learning outcomes. Those who are skilled at self-learning more positively evaluate their learning skills, and thereby, through self-determination, set more optimistic learning goals (Deci and Ryan, 1985). Moreover, optimistic people are adept at adopting direct actions, plans, and coping strategies in learning, thus improving their academic outcomes (Luthans et al., 2015). Studies have similarly confirmed that students can more effectively observe their own performance and assess their own targeted progress when they participate in learning activities with a positive goal and a desire to achieve goals (Schunk, 1990). More positive emotions, accompanied by positive psychological implications, are induced in students with strong self-directed learning skills as their satisfaction with their targeted progress increases. In sum, students with greater self-directed learning skills are more likely to generate optimism as psychological capital. Furthermore, according to the theory of psychological capital, optimism pertains to positive emotions and can motivate an individual to achieve higher performance (Luthans and Youssef, 2007; Luthans et al., 2015). Research has revealed that, compared with individuals with high optimism, those with low optimism are less capable of adopting direct actions, planning, and coping strategies to manage and relieve the stress caused by life events when perceiving stress, thus lowering the efficiency of their work and learning (Brissette et al., 2002). Optimism can predict favorable academic results, and those who are optimistic possess sufficient social adaptability (Alkhedr, 1999; Dougall et al., 2001). El-Anzi and Owayed (2005) also highlighted that academic performance can be improved through optimism. Therefore, on the basis of the abovementioned theoretical perspectives and research findings, Hypothesis 2 was formulated as follows:

H2: Optimism plays a mediating role in the influence of self-directed learning on academic performance.

### Self-Directed Learning, Mental Health, and Academic Performance

Mental health is defined as a state of well-being; in a state of mental health, people feel relaxed and can adapt to circumstances and thoroughly reach their psychophysical potential (Zhang et al., 2013). Social ecological systems theory (Bronfenbrenner, 1992) suggests that difficulties related to people’s intimacy and communication in school and family life as well as to people’s emotional response and behavioral control may cause mental health problems such as self-isolation and alienation. A healthy mentality provides learners with a favorable mental environment, and this positive mental state determines whether individuals can realize their academic potential (Maslow, 1954). In self-determination theory, environmental factors are also the basis of an individual’s self-directed learning behavior (Deci and Ryan, 1985). However, spontaneous learners can adopt active learning behaviors involving self-monitoring to adjust their psychological state and improve their academic outcomes more effectively; those with stronger self-directed learning skills exhibit a more positive and active learning status (Deci and Ryan, 1985; Luthans and Youssef, 2007). Such positive learning behavior is more consistent with the expectations of society, school, and parents, and the learner is more likely to obtain affirmation from teachers and parents, thus sustaining their relaxed psychological state (Tuffin et al., 2001) and achieving learning outcomes. Therefore, students skilled in self-directed learning exhibit behavior that is consistent with social expectations and receive social support to maintain their mental health (Tuffin et al., 2001; Hefner and Eisenberg, 2010). Favorable mental health provides learners with a positive psychological environment as support, which affects whether they can realize their academic potential (Maslow, 1954). According to studies on learning and well-being, a self-monitoring learning style influences Students’ perceptions of successes and improves their mental state (Field, 2009; Teal et al., 2015). Byrd and Mckinney (2012) reported that mentally healthy college students have greater advantages in academic performance in terms of interpersonal adaptation, communication, and acquiring cognitive ability relative to those with lower levels of mental health. Related studies have also revealed that mental health significantly and positively predicts Students’ academic performance (Scheier and Carver, 1985; Alkhedr, 1999; Albeg and Castro-Olivo, 2014; Cazan and Schiopca, 2014). Combining the aforementioned theories and research results, Hypothesis 3 was formulated as follows:

H3: Mental health plays a mediating role in the influence of self-directed learning on academic performance.

### Self-Directed Learning, Optimism, Mental Health, and Academic Performance

According to self-determination theory, a direct positive relationship exists between self-determination behavior and performance (Deci and Ryan, 1985). However, the relation of self-determination learning with learning outcomes may be jointly mediated by optimism and mental health. According to psychological capital theory, optimism is a critical positive resource in personality (Luthans et al., 2015). This positive personality tendency represents an effective internal ability for self-reparation and enhancement, acting as a buffer in the process of the psychological stress response and helping people resist mental illness (Chang, 2002; Gustavsson-Lilius et al., 2007). Zhang et al. (2011) revealed that optimism and psychological capital can help people withstand the impact on mental health resulting from the pressure of learning. Similarly, research in other fields has revealed that the mental health of optimistic patients with cancer seems to be superior to that of unoptimistic ones (Schou et al., 2004). Optimism negatively predicts psychological problems including suicidal tendencies and negative emotions (Yu and Zheng, 2011). Optimism is regarded as a predictor of mental health, supporting the notion that optimism and mental health may act as chain-mediators but not in mutually independent mediation roles. When viewed collectively, self-determination theory and psychological capital theory suggest that people with high-level self-learning skills more positively assess their learning skills, thereby inducing positive and optimistic emotional experiences. As positive psychological capital, optimism can help people resist mental health problems caused by stress (Deci and Ryan, 1985; Chang, 2002; Gustavsson-Lilius et al., 2007; Zhang et al., 2011; Luthans et al., 2015). Furthermore, needs theory suggests that a healthy mental state in learners determines whether they can successfully realize their academic potential (Maslow, 1954; Elliot and Dweck, 1989). Studies have also confirmed that people with strong self-learning skills exhibit more positive self-assessment, leading to optimistic emotional perception in such learners (Alkhedr, 1999; El-Anzi and Owayed, 2005), and those with optimistic positive expectations exhibit superior mental health (Chang, 2002; Schou et al., 2004; Gustavsson-Lilius et al., 2007; Zhang et al., 2011; Luthans et al., 2015). Favorable mental health also predicts higher academic performance (Kantomaa et al., 2010; Albeg and Castro-Olivo, 2014; Bostani et al., 2014; Agnafors et al., 2020). Therefore, this study posited that, for Wuhan college students, although optimistic learning attitudes and experiences result from spontaneous learning, such optimistic positive emotions may in turn affect mental health, thereby providing support for academic performance through a healthy psychological state. Thus, Hypothesis 4 was formulated as follows:

H4: Optimism and mental health play chain-mediating roles in the influence of self-directed learning on academic performance.

### Hypothetical Model

**FIGURE 1:**
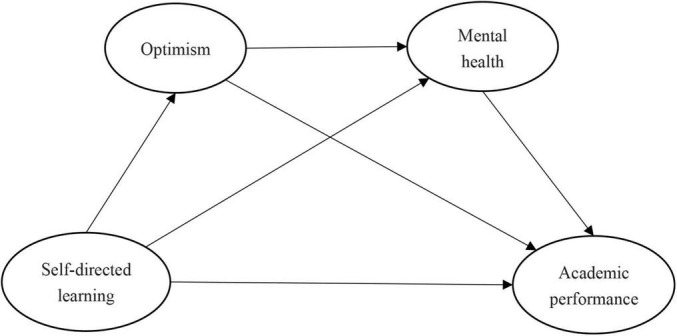
Hypothetical model.

This study’s hypothetical model (Figure 1) was developed with reference to relevant theories and studies. In the model, self-directed learning is assumed to positively predict the academic performance of college students in Wuhan, and optimism and mental health are the two chain-mediating factors in this relationship.

## Materials and Methods

### Materials

Four constructs were investigated through independent scales; each item was scored using a 5-point Likert scale from 1 (*strongly disagree*) to 5 (*strongly agree*). The constructs and scales are described in the following section.

**Self-directed learning** is a learning approach that requires the learner to establish learning goals and a learning plan in addition to employing active self-management and learning self-evaluation (Holec, 1979). The self-directed learning scale contains five items related to self-directed learning ability (Hung et al., 2010). An example item is as follows: “I carry out my own study plan.”

**Optimism** plays a positive role in people’s adaptation to stress and coping under challenging conditions. Optimism refers to an expectation of a favorable future outcome (Scheier and Carver, 1985). Eight items are included in the optimism scale (Scheier and Carver, 1985). An example item is as follows: “In uncertain times, I usually expect the best.” After confirmatory factor analysis (CFA), three items for which the factor loading was considerably below 0.50 were excluded, and the remaining five items were used in the modeling (Kline, 2010).

**Mental health** refers to the positive state of an individual’s well-being. Those who are in this state have favorable adaptability and can employ their full mental potential (Zhang et al., 2013). The mental health scale contains six items (Zhang et al., 2013). An example item is as follows: “I enjoy my life.” In this study, after CFA, one item with factor loading < 0.50 was excluded, and the remaining five items were used in the modeling (Kline, 2010).

**Academic performance** in the present study refers to the comprehensive ability of Chinese college students in terms of learning cognitive, communicative, and interpersonal skills required for their future endeavors (Li et al., 2016). The academic performance scale contains 14 questions in three parts: learning cognitive, communicative, and interpersonal abilities (Yang and Swekwi, 2021). An example item is as follows: “I can use the knowledge that I learned flexibly.”

### Participants

Because pandemic-related regulations in China would have severely hampered in-person surveying, an online questionnaire was employed for purposive sampling. Data were collected from 473 college students (age: 18–25 years) from three universities in Wuhan between April 1 and 15, 2020. All three universities, with the same educational goal, attempted to equip students for self-directed learning and implemented extensive and long-term training related to self-directed learning. To ensure students from all majors would be recruited, university counselors assisted the investigators in recruiting participants through the use of WeChat groups that included dormitory residents. After acknowledging their understanding of the study objectives, all respondents provided signed online informed consent. Respondents were informed that their data would remain confidential.

In total, 500 completed questionnaires were received, with 473 (94.6%) remaining after invalid questionnaires were excluded. Among the valid responses, 219 (46.3%) were from men and 254 (53.7%) were from women; 238 (50.3%) respondents were only children and 235 (49.7%) had siblings; 162 participants (34.2%) were freshmen, 79 (16.7%) were sophomores, 152 (32.1%) were juniors, and 80 (16.9%) were seniors (Table 1).

**TABLE 1:** Demographic distribution of the sample.

Variables	Groups	N	%
Sex	Male	219	46.3
	Female	254	53.7
Only-child	No	235	49.7
	Yes	238	50.3
Age	18–25 years old	473	100
Grade	Freshman	162	34.2
	Sophomore	79	16.7
	Junior	152	32.1
	Senior	80	16.9

### Analytical Method

AMOS and SPSS were employed for statistical analyses, and the hypothetical model was assessed through structural equation modeling (SEM). The measurement and structural models were also verified (Bollen, 1989; Schumacker and Lomax, 2004; Byrne, 2010; Kline, 2010). CFA was employed to test a reasonable measurement model. The association of the Students’ data with the measurement mode was verified by using maximum-likelihood-estimated model parameters and fit indices obtained as statistical indicators. The statistical indicators were factor loadings per factor and measurement errors. Nine indicators of fit were calculated: normed chi-square (χ^2^/df), root mean square residual (RMR), root mean square error of approximation (RMSEA), goodness-of-fit index (GFI), normed fit index (NFI), comparative fit index (CFI), Tucker–Lewis index (TLI), parsimonious normed fit index (PNFI), and HOELTER0.05 (Bollen, 1989; Schumacker and Lomax, 2004). Furthermore, to confirm the reliability and validity of the measurement model, the Cronbach’s α reliability coefficient, average variance extracted (AVE), and composite reliability (CR) were used. Harman’s one-factor test was employed for common method variance (CMV) verification of the study variables (Podsakoff et al., 2003). A bootstrap method was used to verify the mediator roles.

### Reliability and Validity

To evaluate the consistency of variables, Cronbach’s α was used. The relevant values were 0.79, 0.84, 0.82, and 0.91 in the self-directed learning scale, optimism scale, mental health scale, and academic performance scale, respectively, indicating high consistency in the measurement results. For model calibration, this study deleted four items with factor loadings much lower than 0.50. Thus, 29 valid items remained (Table 2). As a final step, the item–objective congruences of the four scales were assessed by five experts; all items in these scales were determined to have high content validity. The model fit indices are presented in Table 3, and the AVE and CR values pertaining to the measurement model and variable correlation matrix are listed in Table 4. The 95% confidence intervals of the correlation coefficients for 2,000 bootstrap replications were calculated for discriminant validity (Torkzadeh et al., 2003).

**TABLE 2:** Questionnaire items employed in this research.

Var	Questionnaire items	M	SRW	Error variance	SE
SL	Q1: I carry out my own study plan.	3.49	0.70	12.55***	
	Q2: I seek assistance when facing learning problems.	3.62	0.54	14.16***	0.07
	Q3: I manage my time well.	3.22	0.76	11.48***	0.09
	Q4: I set up my learning goals.	3.53	0.74	11.84***	0.08
	Q5: I have high expectations for my learning performance.	3.99	0.53	14.23***	0.07
OP	Q1: In uncertain times, I usually expect the best.	3.50	0.63	13.89***	
	Q2: If things can go wrong, I generally end up fine.	3.24	0.63	3.92***	0.09
	Q3: I always look on the bright side of things.	3.50	0.81	11.15***	0.08
	Q4: I’m always optimistic about my future.	3.53	0.75	12.43***	0.08
	Q5: Things always work out the way I want them to.	3.34	0.73	12.86***	0.08
MH	Q1: I enjoy my life.	3.76	0.65	13.59***	
	Q2: I feel that my life is meaningful.	3.89	0.61	13.96***	0.09
	Q3: I can concentrate (for example, thinking, studying, and remembering) on what I want to do.	3.70	0.73	12.64***	0.09
	Q4: I can accept my appearance.	3.72	0.70	13.06***	0.10
	Q5: I am satisfied with myself.	3.61	0.75	12.29***	0.10
LCA	Q1: I can use the knowledge that I learned flexibly.	3.48	0.74	13.27***	
	Q2: I can easily understand what the teacher said in class.	3.47	0.78	12.63***	0.07
	Q3: I can quickly grasp the key to solving a problem.	3.48	0.84	11.16***	0.07
	Q4: I always understand new knowledge and new skills quickly.	3.51	0.81	12.10***	0.07
CA	Q1: I can communicate clearly with people.	3.68	0.72	13.35***	
	Q2: I know how to change the subject in conversations and can master basic talking points.	3.57	0.73	13.22***	0.07
	Q3: I am good at listening and don’t like to interrupt others.	3.77	0.58	4.49***	0.07
	Q4: I can communicate with others face to face.	3.85	0.68	13.84***	0.06
	Q5: I am willing to take the initiative to communicate with others.	3.68	0.71	13.53***	0.07
IA	Q1: I always take the initiative to help other classmates.	3.73	0.75	12.67***	
	Q2: I can take care of other classmates very well.	3.70	0.73	12.92***	0.06
	Q3: In diverse situations, I can control my behavior effectively.	3.87	0.69	13.44***	0.06
	Q4: I cooperate very well with my classmates.	3.82	0.73	12.89***	0.06
	Q5: I get along well with other people.	3.94	0.73	12.94***	0.06

M, mean; SRW, standardized regression weights; SE, standard error; SL, self-directed learning; OP, optimism; MH, mental health; LCA, learning cognitive ability; CA, communicative ability; IA, interpersonal ability. ***p < 0.001.

**TABLE 3:** CFA results of the model.

Model fit	Standard	Results
X^2^/df	<3	2.48
RMR	<0.08	0.04
RMSEA	<0.08	0.06
GFI	>0.85	0.88
CFI	>0.9	0.92
NFI	>0.9	0.90
TLI	>0.9	0.91
PNFI	>0.5	0.78
HOELTER0.05	>200	215

**TABLE 4:** CR and AVE pertaining to the measurement model and variable correlation matrix.

Var	SL	OP	MH	LCA	CA	IA
SL	0.80 (0.44)					
OP	0.56 (0.46–0.65)	0.84 (0.51)				
MH	0.67 (0.57–0.76)	0.73 (0.66–0.80)	0.82 (0.48)			
LCA	0.72 (0.64–0.79)	0.70 (0.61–0.79)	0.70 (0.61–0.78)	0.87 (0.63)		
CA	0.58 (0.46–0.67)	0.72 (0.64–0.79)	0.74 (0.66–0.81)	0.77 (0.69–0.84)	0.82 (0.47)	
IA	0.45 (0.34–0.56)	0.57 (0.48–0.65)	0.64 (0.54–0.73)	0.58 (0.47–0.67)	0.86 (0.79–0.93)	0.85 (0.52)

*The diagonal numbers are CR (AVE). The lower diagonal numbers denote the coefficients of correlation between two variables and 95% confidence intervals of correlation coefficients. CR, composite reliability; AVE, average variance extracted; SL, self-directed learning; OP, optimism; MH, mental health; LCA, learning cognitive ability; CA, communicative ability; IA, interpersonal ability.*

The normality assessment revealed a Mardia coefficient of 286.14, which was lower than *N* × (*N* + 2) = 899, with *N* being the number of questionnaire items; the absolute skewness and kurtosis values for the 29 items ranged from 0.04 to 0.61 and from 0.01 to 0.72, respectively. The results met the standard for the absolute value of skewness and kurtosis (both < 2; Curran et al., 1996), indicating normally distributed data.

**FIGURE 2:**
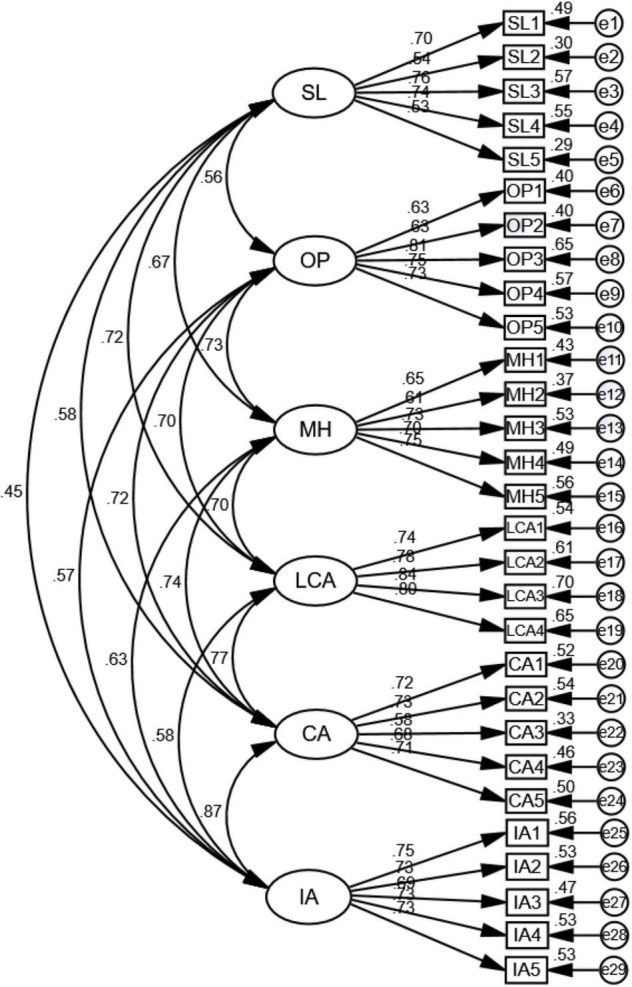
Measurement model.

CFA of the measurement model revealed an absence of negative error variances; moreover, all variances were significant. The factor loadings were all > 0.50 and < 0.95 (Figure 2), with no large standard errors (Table 2; Fornell and Larcker, 1981; Bagozzi and Yi, 2012). Thus, the measurement model was reasonable.

The model fit the data from the Wuhan students reasonably well, with the following fit index values: χ^2^/df=2.48, RMR = 0.04, RMSEA = 0.06, NFI = 0.90, CFI = 0.92, GFI = 0.88, TLI = 0.91, PNFI = 0.78, and HOELTER0.05 = 215 (Table 3; Bollen, 1989; Schumacker and Lomax, 2004).

The measurement model’s convergent validity and discriminant validity were subsequently assessed; its CR was > 0.60 and AVE was > 0.40 (Slater et al., 2007). Moreover, the CR and AVE of all variables were 0.80–0.87 and 0.44–0.63, respectively (Table 4). These results confirmed that the measurement model was acceptable and that it had convergent validity. The 95% confidence intervals of the correlation coefficients with 2,000 bootstrap replications were calculated; none of the confidence intervals in the lower and upper parameters included 1 (Table 4). Thus, the variables had discriminant validity (Torkzadeh et al., 2003).

Finally, because of epidemic prevention and control requirements, this study employed an online questionnaire to limit CMV (Peng et al., 2006). For CMV verification of the study variables, Harman’s one-factor test was employed. Exploratory factor analysis was conducted for all 29 items included in the scales of this study, followed by testing of the unrotated factor analysis results. According to the results, the Kaiser–Meyer–Olkin statistic was 0.95, greater than the threshold of 0.80. The Bartlett test of sphericity also yielded a significant result (*p* < 0.001). The explanatory power of the first factor was 37% (threshold value: 50%), indicating that this study had no severe CMV problem (Podsakoff et al., 2003).

## Results

### Structural Model

**FIGURE 3:**
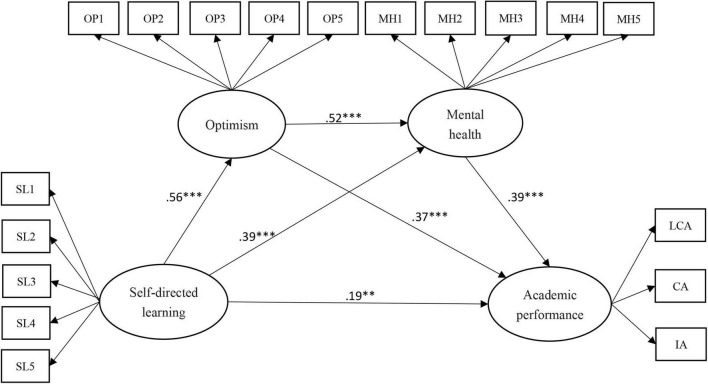
Structural model. **p < 0.01, ***p < 0.001.

SEM of the second-order model was used to obtain a structural model for examining the chain-mediating effect of two mediating variables, optimism and mental health, on the relation of self-directed learning with academic performance (Figure 3). The 29 items in the structural model had standardized regression coefficients of 0.55–0.84. The structural model exhibited a reasonable fit: χ^2^/df = 2.69, RMR = 0.05, RMSEA = 0.06, NFI = 0.90, CFI = 0.91, GFI = 0.86, TLI = 0.90, PNFI = 0.78, and HOELTER0.05 = 207 (Table 5; Bollen, 1989; Schumacker and Lomax, 2004).

**TABLE 5:** Fit of the structural model.

Model fit	Standard	Results
X^2^/df	<3	2.69
RMR	<0.08	0.05
RMSEA	<0.08	0.06
GFI	>0.85	0.86
CFI	>0.9	0.91
NFI	>0.9	0.90
TLI	>0.9	0.90
PNFI	>0.5	0.78
HOELTER0.05	>200	207

### Direct Effect Analysis

As revealed in Figure 3, self-directed learning had positively directly influenced the college Students’ academic performance (γ = 0.19, *p* < 0.01), and 32% of the variation in academic performance could be explained by self-directed learning. The 95% confidence interval of the aforementioned direct effect, as tested using the bias-corrected percentile bootstrap method, was 0.03–0.33 (Table 6); that the confidence interval did not include 0 indicated a direct effect, highlighting self-directed learning as a partial predictor of academic performance. Thus, Hypothesis 1 was confirmed.

**TABLE 6:** Bootstrap path effect analysis.

Effect	Path	Estimate	95% confidence interval	
Direct effect	SL→AP	0.19**	0.029	0.333
Indirect effect	SL→OP→AP	0.21**	0.104	0.247
	SL→MH→AP	0.15**	0.072	0.197
	SL→OP→MH→AP	0.11**	0.055	0.149
Total indirect effect	SL→AP	0.47**	0.373	0.589
Total effect	SL→AP	0.66**	0.545	0.766

**p < 0.01. SL, self-directed learning; OP, optimism; MH, mental health; AP, academic performance.

### Indirect Effect Analysis

According to the mediation model diagram constructed on the basis of the hypotheses of this study, three indirect effect paths existed between self-directed learning and academic performance.

First, self-directed learning directly positively influenced optimism (γ = 0.56, *p* < 0.001), and optimism exhibited a direct positive effect on academic performance (γ = 0.37, *p* < 0.001), with both of these effects being significant. The indirect impact through optimism was of size 0.21 (γ = 0.56 × 0.37, *p* < 0.01), with γ referring to the outcome of the path values of Hypothesis 2 (Figure 3). The indirect influence of this path was determined using bias-corrected percentile bootstrapping with the 95% confidence interval; the resulting interval was 0.10–0.25, which excluded 0, indicating that the mediation effect of optimism in the first path was significant (Table 6); thus, Hypothesis 2 was confirmed and indicated a partial mediation because both direct and indirect effects were observed.

Second, self-directed learning exhibited a direct positive impact on mental health (γ = 0.39, *p* < 0.001), and mental health positively directly influenced academic performance (γ = 0.39, *p* < 0.001), with both effects being significant. The indirect effect through mental health was of size 0.15 (γ = 0.39 × 0.39, *p* < 0.01), with γ referring to the outcome of the path values of Hypothesis 3 (Figure 3). The bias-corrected percentile bootstrap method was used to assess the indirect influence of this path with the 95% confidence interval; the resulting confidence interval was 0.07–0.20, which excluded 0, indicating that mental health had a significant mediating effect in the second path (Table 6); thus, Hypothesis 3 was confirmed and indicated a partial mediation because both direct and indirect effects were present.

Third, self-directed learning had an effect on optimism (γ = 0.56, *p* < 0.001), optimism exhibited a direct positive effect on mental health (γ = 0.52, *p* < 0.001), and mental health positively influenced academic performance (γ = 0.39, *p* < 0.001), with all effects being significant. The indirect impact of self-directed learning on academic performance by means of two chain-mediating variables, optimism and mental health, was of size 0.11 (γ = 0.56 × 0.52 × 0.39, *p* < 0.01), with γ indicating the outcome of the path values of Hypothesis 4 (Figure 3). The bias-corrected percentile bootstrap method was used to assess the indirect influence of this path. The 95% confidence interval was 0.06–0.15, which excluded 0, indicating that the chain-mediated effects of mental health and optimism in the third path were significant (Table 6); thus, Hypothesis 4 was confirmed and reflected partial chain mediation because both direct and indirect effects were observed.

### Total Effect Analysis

According to the preceding analysis (Table 6), the direct effect of self-directed learning on academic performance was of size 0.19 (*p* < 0.01). Self-directed learning’s total indirect impact on academic performance was obtained by summing the effects of three indirect paths: 0.21 + 0.15 + 0.11 = 0.47 (*p* < 0.01). The 95% confidence interval of the indirect effect was 0.37–0.59, which excluded 0, thus confirming the mediating effect. The model’s total effect was 0.19 + 0.47 = 0.66 (*p* < 0.01). The 95% confidence interval of the bootstrap method was used for assessing the total effect, with the resulting interval being 0.55–0.77, which does not include 0; the total effect thus existed. Because total, direct, and indirect effects were present, the structural model was partially mediated; moreover, the three variables—self-directed learning, optimism, and mental health—explained 71.5% of the variation in academic performance.

## Discussion

Hypothesis 1 was verified by the results, thus confirming that Wuhan college Students’ academic performance could be significantly positively predicted by their self-directed learning; this result is consistent with those of related studies (Zhou et al., 2010; Kim, 2017; Oducado, 2021). However, the link between these two variables was not straightforward in the current study. The results confirmed Hypotheses 2 and 3 and verified that optimism and mental health partially mediate the impact of self-directed learning on Wuhan college Students’ academic performance. Furthermore, the results confirmed Hypothesis 4 and revealed that optimism and mental health, as two associated mediators, chain-mediate a partial relationship of self-directed learning with academic performance.

Self-determination theory suggests that spontaneous learning behavior can lead to more favorable learning outcomes (Deci and Ryan, 1985). The theoretical view has been confirmed in relevant studies into self-directed learning and academic performance (Zhou et al., 2010; Kim, 2017; Oducado, 2021), with the current results also verifying this perspective. In a context where online teaching has become normalized in most colleges and universities, Wuhan college Students’ self-directed learning skills appeared particularly crucial. The degree to which students can self-direct their education not only their performance in college but also ensures the smooth development of online teaching (Zhang W. et al., 2020; Zhang X. et al., 2020; Zhang Z. et al., 2020). However, self-directed learning does not contribute to academic performance in a direct and simple manner, a result that differs from those reported in studies into the same topic.

In this study, self-directed learning was revealed to contribute indirectly to academic performance through the mediating effects of optimism and mental health. First, the results indicated that self-directed learning not only directly predicts academic achievement (Zhou et al., 2010; Kim, 2017; Oducado, 2021) but also predicts academic achievement through optimism. A part of the self-directed learning–academic performance relationship appears in an indirect process associated with optimism; self-directed learning makes a significant positive contribution to optimism (Elliot and Dweck, 1989), thereby becoming a significant positive predictor of Wuhan college Students’ academic performance (El-Anzi and Owayed, 2005; Wang et al., 2011; Bai and Xie, 2018). According to self-determination theory and psychological capital theory, the indirect relationship suggests that self-determination behaviors can increase the strength of positive and optimistic emotions. Furthermore, optimism is described as a trait pertaining to psychological capital and that motivates individuals to attain superior outcomes and performance (Deci and Ryan, 1985; Luthans et al., 2015). Therefore, individuals with stronger self-learning skills may have more confidence and positive expectations regarding their learning skills and behavior; they are more likely to experience optimistic emotions and maintain highly effective academic performance. Thus, for Wuhan college students, spontaneous learning may be an approach that can stimulate optimistic emotions and result in positive academic outcomes (Deci and Ryan, 1985; Luthans and Youssef, 2007). Second, the relation of self-directed learning with academic achievement (Zhou et al., 2010; Kim, 2017; Oducado, 2021) is also partially mediated through mental health. The indirect process associated with mental health in the connection of self-directed learning with academic performance suggests that self-directed learning makes a significant positive contribution to mental health (Tuffin et al., 2001; Field, 2009; Chen, 2015; Teal et al., 2015), thereby serving as a significant positive predictor of the academic performance of Wuhan college students (Kantomaa et al., 2010; Bostani et al., 2014; Zhao, 2020). When viewed together, ecological systems theory and self-determination theory suggest that individuals who employ initiative in learning are more likely to meet their own expectations as well as those of their parents and society, thereby obtaining positive feedback and emotional support; such positive support, exchange, and communication protect the learner’s mental health, which in turn provides support through a healthy psychological state for maximizing academic achievement (Maslow, 1954; Bronfenbrenner, 1992; Ryan and Deci, 2000; Luthans and Youssef, 2007). Thus, learning behaviors involving initiative may help Wuhan college students maintain a healthy psychological state, ensuring optimal academic outcomes.

The indirect relation of self-directed learning with academic performance, however, is not only mediated through optimism and mental health, independently. The optimism–mental health association indicated that optimism can serve as a significant positive predictor of mental health (Chang, 2002; Gustavsson-Lilius et al., 2007; Hanssen et al., 2014). The two mediators (optimism and mental health) thus constitute chain mediation and jointly mediate the partial relation of self-directed learning with academic performance among college students in Wuhan. That is, self-directed learning not only directly predicts the academic performance of Wuhan college students but also supports optimism, thus influencing mental health and predicting academic achievement. When regarded from the perspectives of self-determination theory, psychological capital theory, and ecological systems theory, the chain mediation may reveal that spontaneous learning behaviors provide people with a joyful emotional experience; joyful emotions have a positive effect on mental health, and this positive effect provides support through a healthy psychological state to ensure an individual’s academic achievement (Maslow, 1954; Deci and Ryan, 1985; Bronfenbrenner, 1992; Ryan and Deci, 2000; Luthans et al., 2015). The current results, consistent with previously obtained findings, reveal that spontaneous learning behaviors may induce optimistic emotions in learners (Alkhedr, 1999; El-Anzi and Owayed, 2005), and optimistic individuals are more adept at preserving a healthy psychological state to resist academic pressure (Zhang et al., 2011; Hanssen et al., 2014), thereby optimally benefiting learning outcomes (Bostani et al., 2014; Agnafors et al., 2020). The findings for Hypotheses 1–4 demonstrate that self-directed learning is essential to the academic outcomes of Wuhan college students. Online learning has become ubiquitous; thus, self-directed learning is an indispensable learning ability for Chinese college students, enabling them to adapt to online education and produce favorable learning outcomes (Zhang W. et al., 2020; Zhang X. et al., 2020; Zhang Z. et al., 2020). The benefit of self-directed learning for the learning outcomes of Wuhan college students may have greater relevance than merely suggesting that self-directed learning can predict academic performance in this population. In the chain mediation mechanism through which optimistic emotions are induced by self-directed learning, these positive emotions can help stimulate a healthy psychological state, which then affects academic performance. Thus, this chain mediation may reveal the importance of Wuhan college Students’ self-directed learning for motivating them to maximize their achievement while participating in online education.

## Conclusion

This study found that, during online teaching, self-directed learning serves as a significant positive predictor of the academic performance of college students, but it cannot completely explain the academic performance of these students. The relation of self-directed learning with academic performance can be partially chain-mediated through optimism and mental health.

### Suggestions

This research proposes that self-directed learning is an essential learning method that can help Wuhan college students adapt to online education and obtain favorable learning outcomes because of the positive direct and indirect effects prompted by self-directed learning that lead to academic success. On the basis of the importance of self-directed learning for Wuhan college students, this research offers the following two recommendations for administrators of Chinese higher education and student managers:

First, online learning is a safer teaching mode in the current global pandemic context, and self-directed learning can be regarded as a prerequisite for the smooth implementation of online teaching (Zhang W. et al., 2020; Zhang X. et al., 2020; Zhang Z. et al., 2020). This study found that self-directed learning is a predictor of academic achievement. Therefore, Chinese universities (such as those in Wuhan city) should prioritize Students’ self-directed learning skills, help these students develop into self-directed learners, and accommodate online education.

Second, the chain mediation results revealed that students with stronger self-directed learning skills may possess an optimistic mindset that also provides them with a healthy mental state for obtaining positive academic outcomes (Alkhedr, 1999; El-Anzi and Owayed, 2005; Zhang et al., 2011; Bostani et al., 2014; Hanssen et al., 2014; Agnafors et al., 2020). Therefore, the positive indirect effect of self-directed learning on academic performance may also serve as a reminder that higher education in China should follow the concept of constructivist teaching, in which students are encouraged to actively explore and discover knowledge as well as keenly construct the meaning of the knowledge they acquire (Piaget, 1980). Through this approach, students may become more optimistic and active by means of self-directed learning, thereby maintaining their mental health, resisting academic stress, and obtaining more favorable learning outcomes. Thus, this research recommends that counselors serve as guides for students in colleges and universities in China (Ministry of Education of the People’s Republic of China, 2017), training these students in self-directed learning and helping them become active learners with an optimistic attitude and relaxed mental state during online teaching.

### Limitations and Future Directions

Only the self-directed learning situation of Wuhan college students was investigated herein because Wuhan is the most representative area of where the pandemic first broke out in China. However, online teaching has also been applied in other countries and throughout China. Future research may compare the results obtained in diverse regions or countries.

Because of the restrictions related to pandemic prevention and control in China, this study collected data solely through an online survey questionnaire. In the present investigation of Wuhan college students, a group with long-term online learning experience because of the COVID-19 pandemic, only the survey method was employed and relevant behavioral details and manifestations of the interviewees could not be obtained. Therefore, if future policy permits, follow-up research may integrate face-to-face interviews to further supplement the research data.

This was a cross-sectional study, and only the potential relationships between variables could be analyzed using SEM. Future studies may use experimental techniques to examine the connection of self-directed learning with learning performance.

## Data Availability Statement

Other data pertaining to this study are available from the corresponding author upon reasonable request.

## Ethics Statement

Ethical review and approval was not required for the study on human participants in accordance with the local legislation and institutional requirements. The patients/participants provided their written informed consent to participate in this study.

## Author Contributions

JL was the primary author of this research and prepared this article, which involved writing and data analysis. ZH worked as an investigator and a writer’s assistant. DY prepared the research proposal and served as the research advisor for specific matters. JL, DY, and ZH revised collaboratively the manuscript. All authors contributed to the article and approved the submitted version.

## Conflict of Interest

The authors declare that the research was conducted in the absence of any commercial or financial relationships that could be construed as a potential conflict of interest.

## Publisher’s Note

All claims expressed in this article are solely those of the authors and do not necessarily represent those of their affiliated organizations, or those of the publisher, the editors and the reviewers. Any product that may be evaluated in this article, or claim that may be made by its manufacturer, is not guaranteed or endorsed by the publisher.
